# Supplementary figures and images for: Ecm29-Dependent Proteasome Localization Regulates Cytoskeleton Remodeling at the Immune Synapse
Source: Front Cell Dev Biol. 2021 May 13;9:650817. doi: 10.3389/fcell.2021.650817 (PMC8155528; doi:10.3389/fcell.2021.650817)

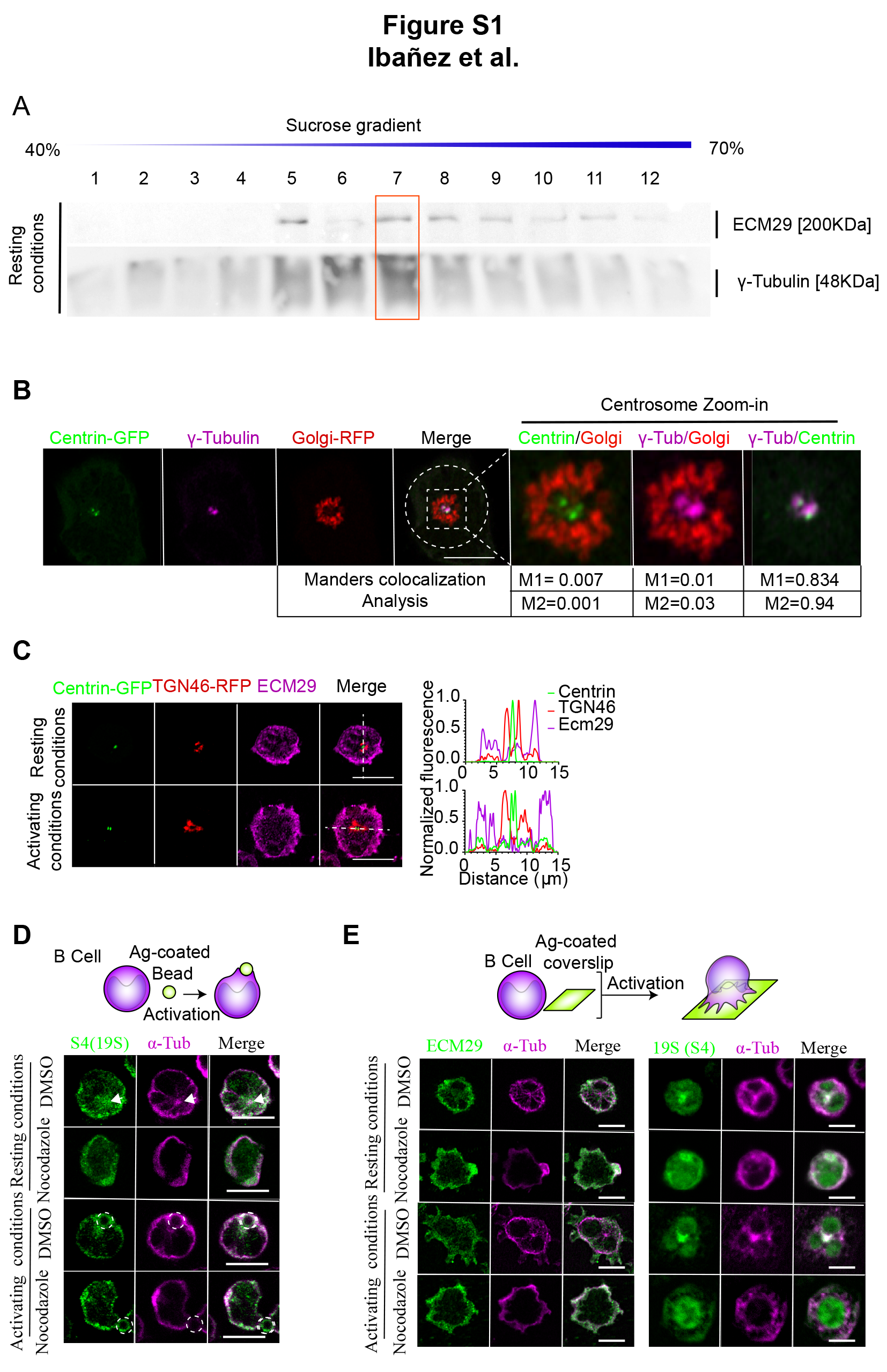

Supplement: Supplementary Figure 1 — Ecm29 cofractionates with the centrosome in B cells. (A) Representative immunoblot of centrosome fractions isolated from B cells in resting conditions, where γ-tubulin (centrosome) and Ecm29 were detected. (B) Representative confocal images of B cells in resting conditions, showing Centrin-GFP, γ-tubulin, and Golgi, and co-localization analysis. (C) Representative confocal images of B cells in resting or activating conditions, showing Golgi, Centrin, and Ecm29, and line-scan analysis of each label across the cell. (D) Representative images of B cells in resting or activating conditions (60 min of activation) with antigen-coated beads, pretreated with DMSO as a control, 30 μM Nocodazole (NocZ) for 30 min. For activated B cells, drug treatment was performed after 30 min of activation. S4(19S RP), α-Tubulin, F-actin, and merge are shown. (F) Representative images of resting B cells or activated on antigen-coated coverslips (60 min of activation) pretreated with DMSO as a control, 30 μM Nocodazole (NocZ) for 30 min. For activated B cells, drug treatment was performed after 30 min of activation. Two sets of staining are shown: (1) S4(19S RP), α-Tubulin, F-actin, merge, and (2) Ecm29, α-Tubulin, F-actin, and merge, are shown. Scale Bar = 10 μm. [file Image_1.tif]

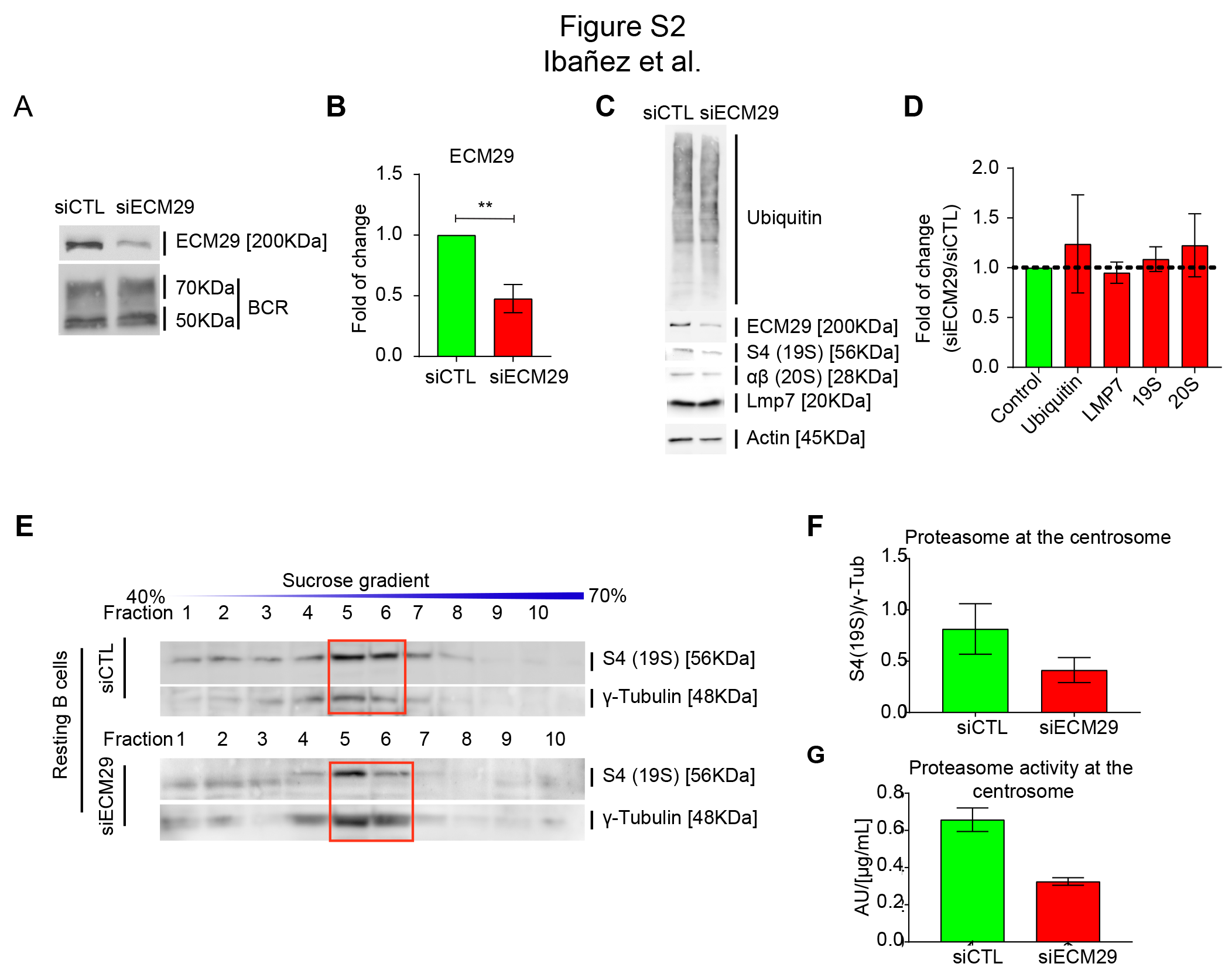

Supplement: Supplementary Figure 2 — Ecm29 silencing does not affect levels of ubiquitylated proteins, but reduces proteasome levels at the centrosome in resting B cells. (A) Representative immunoblot of protein extracts obtained from resting B cells transfected with scrambled siRNA (siCTL) and Ecm29 targeted siRNA (siECM29). Ecm29 and BCR are shown. (B) Quantification Ecm29 levels in (A). N = 7. (C) Representative immunoblot of control and Ecm29-silenced B cells in resting conditions, stained for Ubiquitin, Ecm29, S4 (19S), αβ (20S), LMP7, and actin. (D) Quantification of protein levels in (C). N > 3. (E) Representative immunoblot of centrosome isolated fractions isolated from control and Ecm29-silenced B cells. S4 (19S) and γ-Tubulin are shown. Red rectangle indicates the centrosome-rich fractions. (F,G) Quantification of S4 (19S) protein levels (N = 3) and proteasome activity (N = 2) in centrosome-rich fractions in (E), respectively. ∗∗p < 0.01. Mann–Whitney test was performed for all statistical analyses. Mean with SEM bars are shown. [file Image_2.tif]

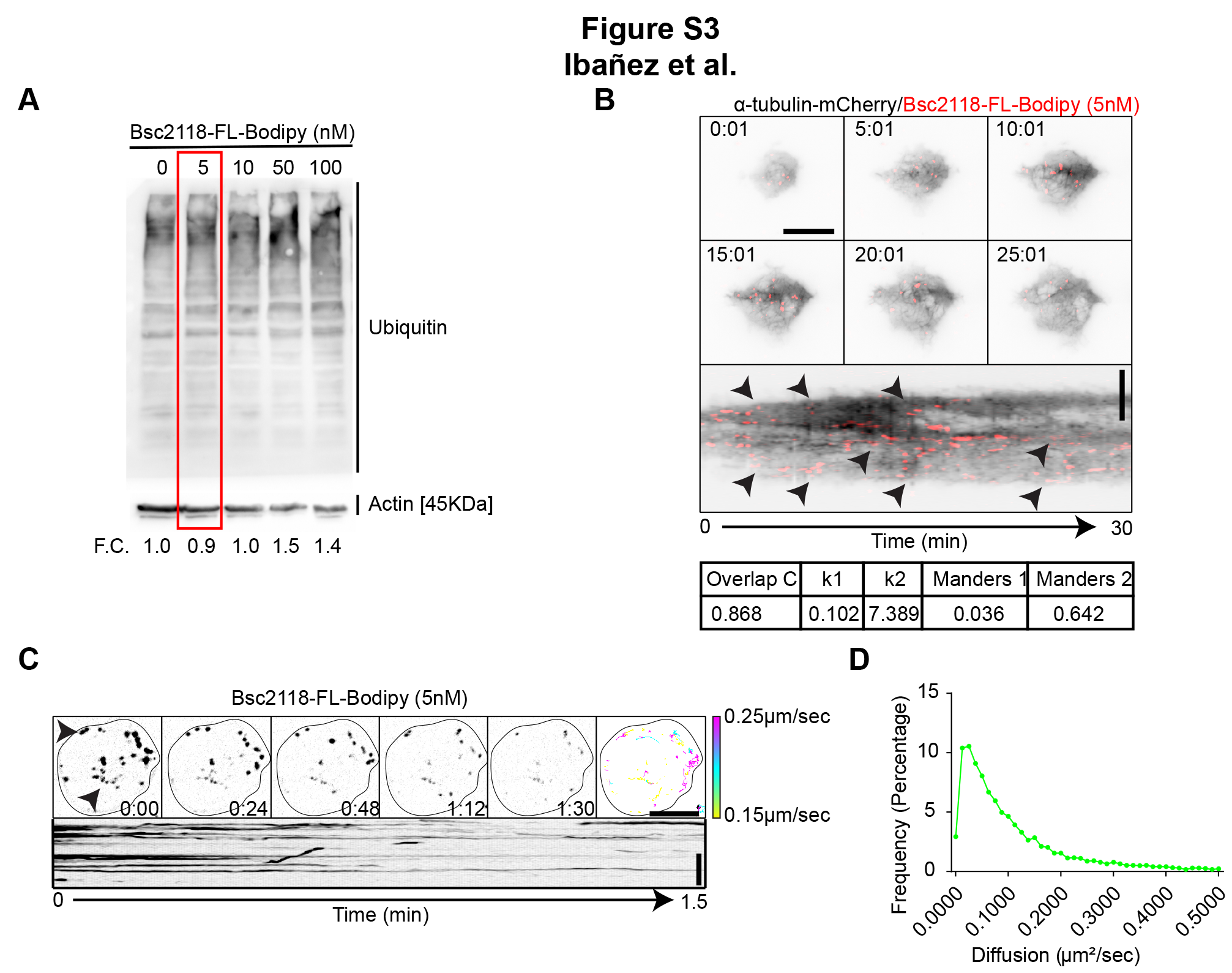

Supplement: Supplementary Figure 3 — Live tracking of the proteasome shows it colocalizes with microtubules and is distributed across the IS. (A) Immunoblot of B cells treated with increasing concentrations of the specific proteasome probe (Bsc2118-FL-Bodipy) for 2 h, ubiquitin and actin labeling are shown. F.C., Fold of Change respect to the control (0 nM of Bsc2118-FL-Bodipy). (B) TIRFM Time-lapse and kymograph of α-Tubulin-mCherry (grayscale) expressing B cells probed with 5 nM Bsc2118-FL-Bodipy (Red). Black arrowheads indicate proteasome positive spots. Below: Table summarizing the colocalization of the proteasome (Bsc2118-FL-Bodipy) and α-Tubulin-mCherry. Overlap coefficient, k1, k2, Manders 1, and Manders 2, are shown. (C) Time-lapse images obtained by TIRFM of B cells labeled for proteasome (grayscale). The accumulation of proteasome tracks (right) is shown. The coldest colors represent the fastest tracks—Kymograph (below). Black arrowheads indicate proteasome positive spots at center and periphery. (D) Histogram of proteasome diffusion coefficient measured in (C). N = 2, Cells > 30. Scale Bar = 10 μm. [file Image_3.tif]

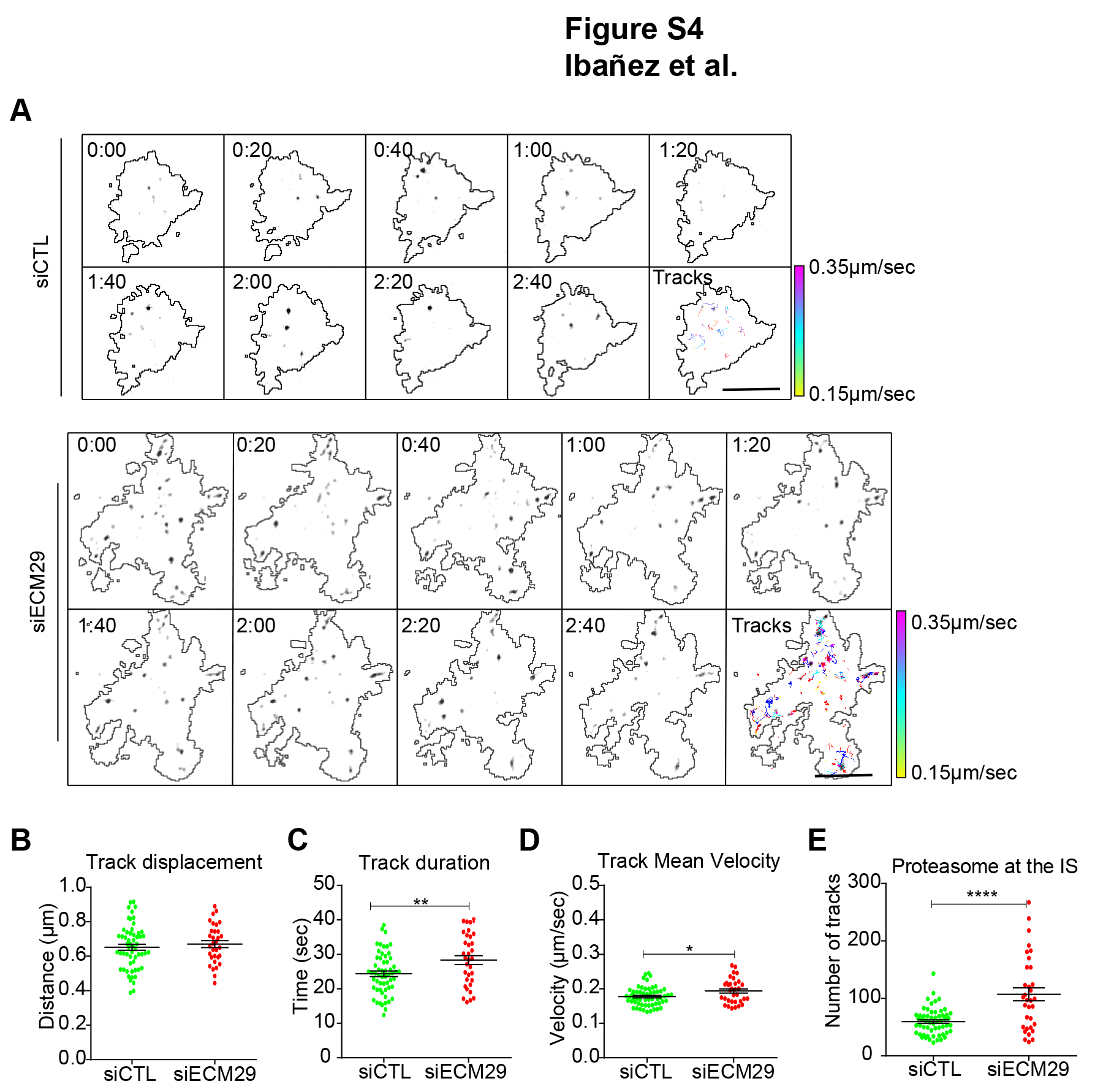

Supplement: Supplementary Figure 4 — Proteasome recruitment and distribution at the IS rely on Ecm29: (A) Representative Time-lapse images by TIRFM of control (siCTL) and Ecm29-silenced (siECM29) B cells after 30 min of activation on antigen-coated coverslips, probed with 5 nM Bsc2118-FL-Bodipy (gray scale). The cell boundary (black line) delimited by the LifeAct-mCherry signal (mask) and accumulation of tracks are shown. The coldest colors represent the fastest tracks. (B–E) Quantification of track displacement, track duration, track mean velocity, and the number of tracks of proteasomes at the IS of control or Ecm29 silenced B cells after 30 min of activation on antigen-coated coverslips. N > 20 Cells. ∗p < 0.05, ∗∗p < 0.01, ****p < 0.0001. Mann–Whitney test was performed. Scale Bar = 10 μm. [file Image_4.tif]

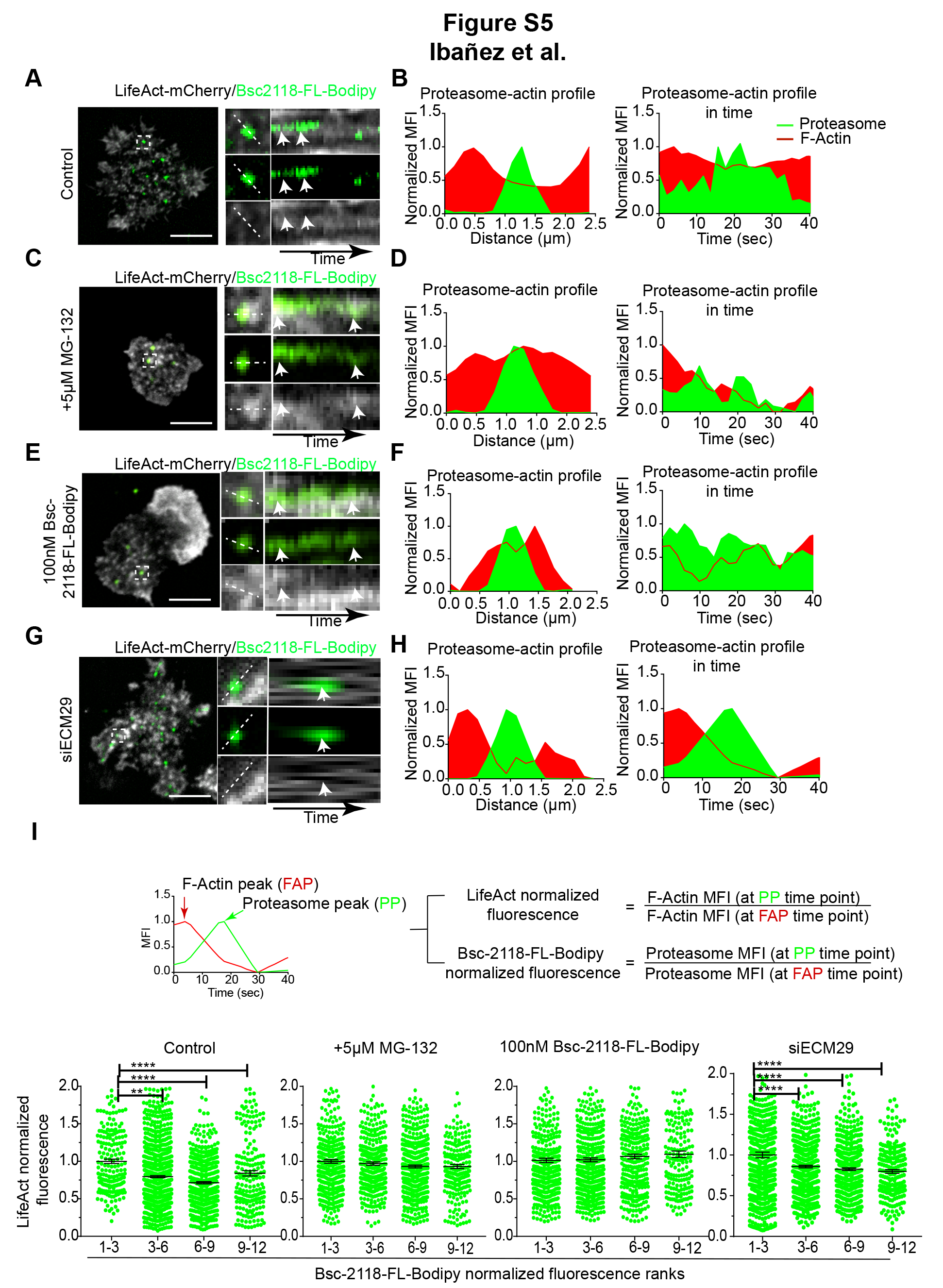

Supplement: Supplementary Figure 5 — Proteasome arrival at the IS negatively regulates actin polymerization. (A,C,E,G) Representative TIRFM images of control, MG-132 pre-treated (5 μM for 1 h), Bsc2118-FL-Bodipy overdosed (100 nM), and Ecm29-silenced (siECM29) B cells. B cells were plated for 30 min onto antigen-coated coverslips and then recorded. LifeAct-mCherry (white) and proteasome (green [Bsc2118-FL-Bodipy]), magnifications proteasome spots (white dashed rectangles), together with their respective kymograph, are shown. White arrowheads indicate the proteasome arrival at the IS close to F-actin structures. (B,D,F,H) Quantification of the proteasome (green) and LifeAct-mCherry (Red) distribution at proteasome positive spots (white dashed line) and their distribution in time (Kymograph) showed on (A,C,D,G), respectively. (I) Scheme illustrating the quantification rationale and results of the quantification of normalized fluorescence intensity correlation between Bsc2118-FL-Bodipy and LifeAct-mCherry, on Bsc2118-FL-Bodipy-positive spots of 1 μm in diameter shown in (A,C,G,E). N > 10. ∗p < 0.05. ∗∗p < 0.001. ∗∗∗p < 0.0005. ****p < 0.0001. Kruskal–Wallis test with Dunn’s test was performed for all statistical analyses was performed. Each dot represents an independent positive Bsc2118-FL-Bodipy spot. Mean with SEM lines are shown. Scale Bar = 10 μm. [file Image_5.tif]

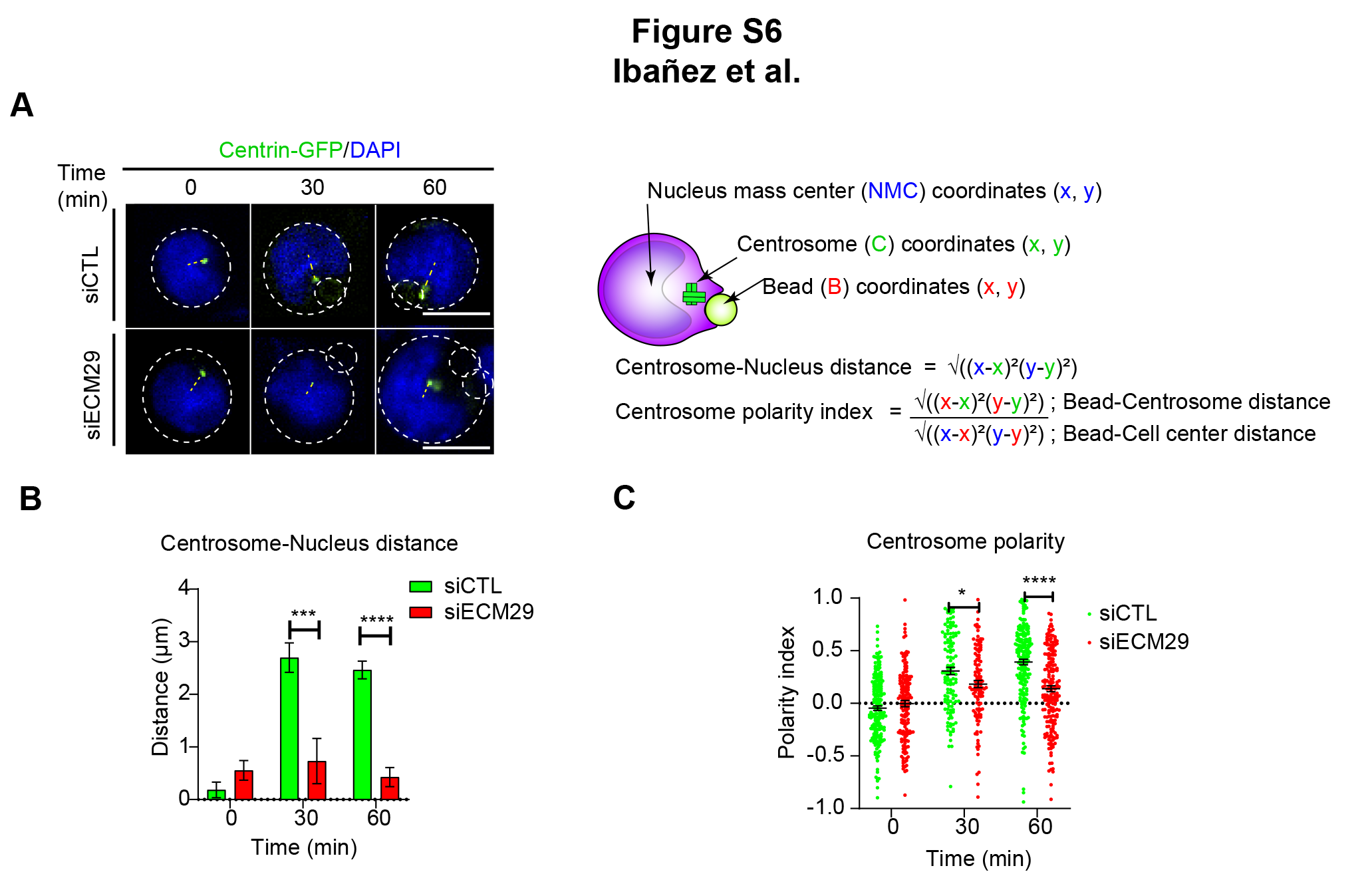

Supplement: Supplementary Figure 6 — Centrosome-nucleus separation and centrosome recruitment to the IS is impaired in Ecm29-silenced B cells: (A) Representative Epifluorescence images of control (siCTL) and Ecm29-silenced (siECM29) B cells, activated with antigen-coated beads for different time points. Centrosome (Centrin-GFP) and nucleus (DAPI) are shown. The dashed line represents the distance between the centrosome-nucleus. A scheme depicting how the cell polarity index and the distance between the centrosome and nucleus were calculated, is shown. (B,C) Quantification of centrosome-nucleus distances and centrosome polarization in (A). N > 3. Cells > 116. Each dot represents an individual measurement (C). Mean with SEM lines (C) and bars (B) are shown. ∗∗∗p < 0.0005. ****p < 0.0001. Kruskal–Wallis test with Dunn’s test was performed. Scale Bar = 10 μm. [file Image_6.tif]
